# CD8+DR+ T-Cells and C3 Complement Serum Concentration as Potential Biomarkers in Thrombotic Antiphospholipid Syndrome

**DOI:** 10.1155/2014/868652

**Published:** 2014-05-29

**Authors:** Elizabeth Sarmiento, Jonathan Dale, Mauricio Arraya, Antonio Gallego, Nallibe Lanio, Joaquin Navarro, Javier Carbone

**Affiliations:** Clinical Immunology Department, Hospital General Universitario Gregorio Marañon, 28007 Madrid, Spain

## Abstract

*Purpose*. To assess complement factors and T lymphocyte activation subset abnormalities in patients with thrombotic antiphospholipid syndrome (APS) as potential biomarkers for development of clinical complications. *Methods*. We assessed C3, C4, factor B concentrations (nephelometry), complement haemolytic functional activity (CH100, radial immune diffusion), and the activation status of CD4+ and CD8+ T-cells (three-colour flow cytometry) in patients with thrombotic APS. Antiphospholipid (aPL) positive patients without APS-related clinical criteria, systemic lupus erythematosus (SLE) patients, and healthy individuals were evaluated as controls. A clinical followup was performed to assess the potential relationship between the immunological parameters and development of APS-related complications. *Results*. Lower concentrations of C3 and higher levels of CD8+DR+ cells were risk factors for development of APS-related complications during followup, including rethrombosis and neuropsychiatric symptoms. Patients with diagnosed thrombotic APS had significantly lower levels of C3, C4, and CH100 as well as higher percentages of activated CD4+DR+ and of CD8+DR+ T-cells than healthy controls but similar to that observed in autoimmune disease controls. *Conclusion*. Lower C3 and C4 complement levels and higher percentages of CD8+DR+ T-cells were observed in thrombotic APS patients. The potential role of these abnormalities as biomarkers of clinical outcome warrants further evaluation in a multicenter study.

## 1. Introduction

Antiphospholipid syndrome (APS) is an autoimmune systemic disease characterized by one or more clinical episodes of venous or arterial thrombosis and obstetric complications, together with antiphospholipid antibodies (aPL) in serum [1]. Clinical complications may occur in these patients during followup. There is a lack of biomarkers to identify patients at risk for development of these clinical complications. To identify a biomarker the first step is the identification of a potential pathogenic role of the biomarker in the evaluated disease. Then the association between the biomarker and clinical outcomes should be assessed.

Activation of complement proteins by pathogen-associated and danger-associated molecular patterns induces generation of distinct effector molecules [2]. An association between aPL-induced morbidity during pregnancy and complement activation has been reported [3–5]. In murine models, complement plays an important role in fetal injury in animals with APS [6]. Serum complement proteins and function are commonly assessed to establish the degree of activation of this system in specific clinical conditions, although they have not been extensively assessed in patients with thrombotic APS [7]. Activation of T-cells has also been reported to play a role in the pathogenesis of specific models of thrombotic disease [8, 9]. Interestingly enough, the most common therapeutic interventions in thrombotic APS, such as warfarin or heparin, have the potential to decrease lymphocyte activation and complement activation, respectively [10, 11]. Recent research supports a role for the complement system in the regulation of T-cell responses [12]. In this study, we assessed specific complement system parameters and lymphocyte activation subsets to assess their relationship with thrombotic APS and to set up the hypothesis about their potential role as biomarkers of clinical outcome in a prospective study.

## 2. Patients and Methods

### 2.1. Patients

The present study was performed in two parts: the first part was a cross-sectional case-control study to assess the relationship between immunological biomarkers and thrombotic APS; the second part was a prospective follow-up study that used the immunological study as baseline to assess whether the evaluated immunological biomarkers are potential candidates for identifying patients at risk of APS-related complications.

We included 38 patients with thrombotic APS. APS was diagnosed according to the Sydney classification criteria [1]. None of the patients had comorbidities associated with impaired complement levels, including full-blown systemic lupus erythematosus (SLE) or other systemic autoimmune diseases, chronic liver disease, recent opportunistic infections (last 6 months), and suspicion of a complement primary immunodeficiency (previous history of recurrent bacterial infections). The patients' blood was not examined in the acute phase of thrombosis. None of the patients had thrombotic episodes in the previous 3 months. No patients used heparin or its derivatives during the study or in the 3 months before the study; this may have modified complement activation. Most patients were given antithrombotic agents. Arterial events such as stroke, pulmonary embolism, and myocardial infarction were confirmed by computed tomography (CT) scan, magnetic resonance imaging (MRI), or angiography. Deep vein and arterial thrombosis were confirmed by Doppler echography or angiography.

### 2.2. Controls

The control groups comprised 14 aPL positive patients without APS-related clinical criteria, 16 systemic lupus erythematosus (SLE) patients, and 52 healthy individuals. Within aPL positive controls, all of them had autoimmune diseases (Table 1). None of these controls had full-blown SLE. SLE controls did not have clinical flare-ups during the 3 months before the study. None of the SLE patients had any laboratory criteria of the APS syndrome.

A questionnaire was administered to ensure that none of the healthy controls had any disease associated with altered complement levels.

### 2.3. Plasma and Serum Sample Collection

Immunological studies were performed the first time the patients were evaluated at the clinical immunology unit. Blood was extracted by venipuncture and collected into serum or plasma tubes. For serum extraction, the sample was left to coagulate for 30 minutes before being centrifuged at 1800 rpm for 5 minutes.

### 2.4. aPL Determination

The levels of IgG and IgM anti-cardiolipin (aCL) and IgG and IgM anti-*β*2 glycoprotein (*β*2GPI) antibodies were determined using standard ELISA assays (Fresenius, Bad Homburg, Germany). Levels of lupus anticoagulant (LA) were measured using the assays recommended in the guidelines of the International Society on Thrombosis and Haemostasis.

### 2.5. Serum Quantification and Functional Assessment of Complement Proteins

Levels of C3, C4, and factor B were measured using nephelometry with normal ranges of 83–172 mg/dL, 17–51 mg/dL, and 19–50 mg/dL, respectively (Beckman-Coulter, California, USA). CH100 was measured using radial immune diffusion with a normal range of >70 units/mL (Sanofi-Pasteur, Paris, France). These parameters are included in the protocol of all patients who are evaluated for the first time at the clinical immunology unit.

### 2.6. Evaluation of Activated CD4 and CD8 T-Cells

We performed a substudy of 18 patients, 14 aPL positive controls, 10 SLE patients, and 30 healthy controls. This substudy was not included in routine analyses. We previously investigated the association between levels of CD4 and CD8 subsets and obstetric APS [13], unexplained recurrent miscarriage [14], and chronic infectious diseases in patients who develop thrombosis [15]. In addition to the exclusion criteria set out above, the patients were not immunized and did not have active infections during the 6 months before the immunological study. The monoclonal antibodies used were directly conjugated with fluorescein isothiocyanate (FITC; FL1), phycoerythrin (PE; FL2), or peridinin chlorophyll protein (PerCP; FL3). We enumerated T-cell subsets using FITC/PE/PerCP combinations of HLADR/CD38/CD4, HLA-DR/CD25/CD4, CD28/HLADR/CD8, and isotypical controls. Lymphocyte staining was carried out using a whole-blood lysis technique, according to the recommended methodology and quality control procedures. Stained samples were analyzed using a FACScan flow cytometer (Becton Dickinson, San Jose, CA, USA) with CellQuest Pro software (Becton Dickinson).

### 2.7. Clinical Followup

To assess the potential impact of the evaluated abnormalities on clinical outcome, patients were scheduled to return to the clinical immunology unit at 6-month intervals. The revised criteria for the classification of SLE were used [16]. The cognitive symptoms inventory was used in patients with SLE (21-item questionnaire) to screen for difficulties in daily activities involving intermediate memory, concentration, attention, and executive function. Severe cognitive dysfunction was defined as a score ≥2 standard deviation (SD) below the mean in domains of attention, memory, and psychomotor speed, as compared to normative data [17]. To evaluate the inflammatory status of patients and disease controls, C-reactive protein (CRP assessed by nephelometry), rheumatoid factor (RF assessed by nephelometry), and erythrocyte sedimentation rate (ESR) were included. The disease activity status of SLE controls was assessed by the SLE Disease Activity Index (SLEDAI) [18].

### 2.8. Statistics

We compared the means using ANOVA test. A chi-square test was used to compare the frequencies of qualitative variables. Logistic regression was used to assess the relationship between immunological abnormalities and clinical outcome. Spearman's correlation test was used to assess the relationship between variables. We used a database constructed in Microsoft Excel, and the statistical analysis was performed using SPSS.

### 2.9. Ethics

The procedures followed in this study were in accordance with the ethical standards of our hospital ethics committee and with the Helsinki Declaration of 1975. Informed consent was obtained before the performance of the studies.

## 3. Results

### 3.1. Distribution of aPL

The distribution of aPL was as follows: IgG aCL only, *n* = 8 (21.1%); IgG aCL and IgG anti-*β*2GPI, *n* = 7 (18.4%); IgM aCL and IgM anti-*β*2GPI, *n* = 1 (2.6%); triple positivity (LA, aCL, and anti-*β*2GPI positive), *n* = 20 (52.6%); other, *n* = 2 (5.3%).

### 3.2. Serum Complement Levels and Hypocomplementemia in Patients with Thrombotic APS

The demographic and clinical characteristics of patients and controls are shown in Table 1. Patients with thrombotic APS showed significantly lower levels of C3, C4, and CH100 than healthy controls (Table 2). The prevalence of lower levels of C3 and C4 (defined as a concentration below the lower limit of normal) was higher in patients than in healthy controls (Table 2). Factor B concentrations were similar in patients and healthy controls. Only one patient had lower serum concentrations of factor B. Mean serum complement concentrations and prevalence of hypocomplementemia were similar in thrombotic APS patients as compared with aPL positive controls (Table 2). SLE controls disclosed significantly lower C3 concentrations than thrombotic APS patients.

### 3.3. Association between Laboratory and Clinical Abnormalities and Complement Levels

Serum levels of C3 and C4 were significantly lower in APS patients with high titers of IgG or IgM aCL (defined as >80 units, Table 3). Patients with positive LA activity showed significantly lower C3 levels than those with negative activity (87 ± 17 versus 109 ± 30 mg/dL, *P* = 0.014). Patients with triple positivity disclosed significantly lower serum concentrations of C3 than patients without triple positivity (86 ± 18 versus 111 ± 28 mg/dL, *P* = 0.003).

No significant differences were observed between the concentration of the different complement proteins and CH100 when APS patients with positive antinuclear antibodies (ANA, IIF titer >160, *n* = 8) were compared with those without positive ANA, not even when the patients were stratified by anti-DNA antibodies (*n* = 6) (Table 3). Patients with thrombopenia showed significantly lower CH100 levels (Table 3). The presence of risk factors for thrombosis was not associated with complement levels or activity (Table 3).

### 3.4. Activated Lymphocyte Subsets

**Figure 1:**
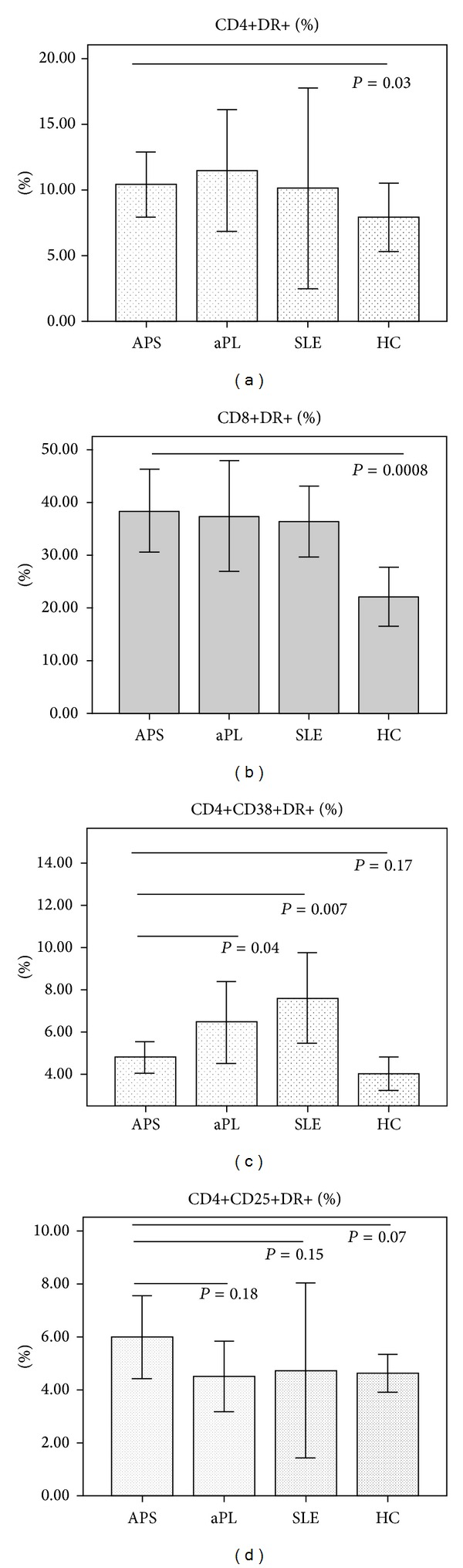
Activated lymphocyte subsets. CD4+ and CD8+ activated T-cell subsets in APS patients and controls. APS: thrombotic APS; aPL: aPL positive controls without APS clinical criteria; HC: healthy controls; SLE: systemic lupus erythematosus controls. Subsets are expressed as percentages over total CD4+ or CD8+ T-cells.

Patients with thrombotic APS had significantly higher percentages of activated CD4+DR+ and CD8+DR+ T-cells than healthy individuals but similar percentages to that observed in aPL positive and SLE controls (Figure 1). There was no association between lymphocyte activation subsets and high titer of aCL, presence of LA, or triple positivity. CD4+CD38+DR+ percentage was significantly higher in autoimmune disease controls as compared with thrombotic APS patients (Figure 1). The percentage of CD4+ cells coexpressing CD38 and HLA-DR increased progressively from thrombotic APS to SLE patients (Figure 1), while the percentage of CD4+ cells coexpressing CD25 and HLA DR tended to be higher in APS patients. Interestingly the ratio of CD4+CD25+DR+/CD4+CD38+DR+ cells was significantly higher in thrombotic APS patients as compared with disease control groups (1.3 ± 0.5, 0.83 ± 0.4, and 0.59 ± 0.36 in APS patients, aPL positive controls, and SLE patients, resp., *P* = 0.004).

No significant correlation was observed between complement levels and T-cell subset percentages (Table 4). C4 values tended to be negatively correlated with activated CD4+DR+ and CD8+DR+ cells (Table 4).

### 3.5. Relationship between Immunological Biomarkers and Clinical Course 

#### 3.5.1. Complement Factors

During the clinical followup performed after the immunological study (mean, 56 months), 11 patients (28.9%) developed APS-related clinical complications (progress to SLE (*n* = 2), epilepsy (*n* = 2), severe cognitive dysfunction (*n* = 4), rethrombosis (*n* = 1), idiopathic thrombocytopenic purpura (*n* = 1), and premature arteriosclerosis [1]). Baseline serum concentrations of C3, C4, and CH100 were significantly lower in patients who later developed complications than in patients who were free of APS-related complications (86 ± 26 versus 107 ± 24 mg/dL (*P* = 0.042), 13 ± 7 versus 21 ± 8 mg/dL (*P* = 0.01), and 21 ± 17 versus 53 ± 16 U/mL (*P* = 0.007), resp.). When we stratified the patients in two groups according to the median values of complement factors, those patients with lower C3 levels (<90 mg/dL) were at higher risk for development of complications (OR 9.99 95% CI, 1.03–97.5, *P* = 0.047).

In APS patients C3 levels disclosed a significantly negative correlation with rheumatoid factor (2-tailed Spearman's correlation coefficient (*R*): −0.56, *P* = 0.036). C4 levels also correlated with rheumatoid factor (*R*: −0.60, *P* = 0.02). CH100 levels negatively correlated with CRP (*R*: −0.69, *P* = 0.006) and with rheumatoid factor (*R*: −0.57, *P* = 0.035). In aPL positive controls we found a positive correlation between C4 levels and serum CRP (*R*: +0.63, *P* = 0.028). In SLE controls we did not find significant correlations between complement levels and rheumatoid factor, CRP, or ESR.

In SLE patients, CH100 levels disclosed a significantly negative correlation with the SLEDAI index (*R*: −0.75, *P* = 0.013).

#### 3.5.2. T-Cell Subsets

**Figure 2:**
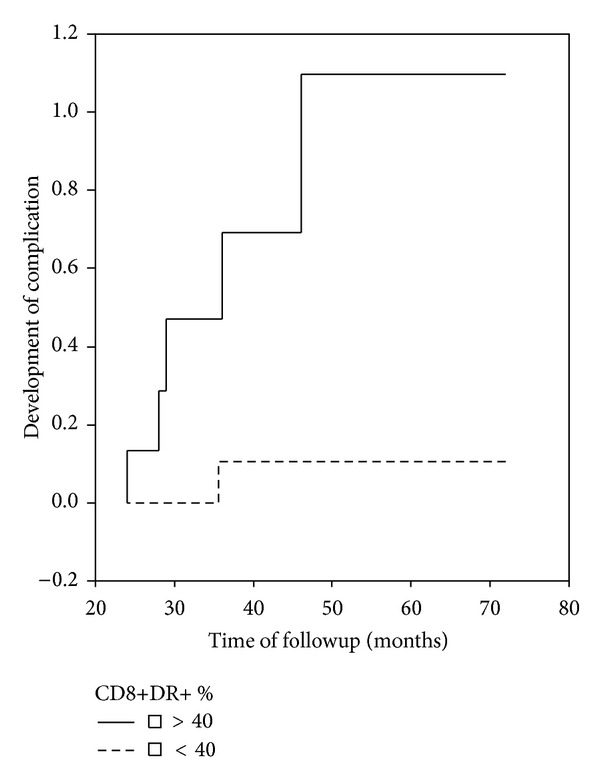
Kaplan Meier curves for development of clinical complications in thrombotic APS patients according to baseline CD8+DR+ percentages. Log Rank 5.43, *P* = 0.019.

During the clinical followup, 6 patients (33.3%) developed APS-related complications (progress to SLE (*n* = 1), epilepsy (*n* = 2), severe cognitive dysfunction (*n* = 1), and rethrombosis (*n* = 2)). This subgroup of patients had significantly higher percentages of CD8+DR+ T-cells (51 ± 20 versus 32 ± 8%, *P* = 0.0007) in the baseline study. When we stratified the patients in two groups according to the median values of lymphocyte subsets, those patients with higher CD8+DR+ levels (>40%) were at higher risk for development of complications (OR 14.9, 95% CI 1.21–185.2, *P* = 0.034) (Figure 2).

None of the evaluated clinical variables (sex (male versus female), age (>50 years versus <50 years), presence of risk factors of thrombosis, presence of triple aPL positivity, and presence of high titer antiphospholipid antibodies) were associated with development of complications (data not shown). In multivariate logistic analysis, higher percentages of CD8+DR+ (>40%) cells remained as a risk factor for development of complications (OR 14.9, 95% CI 1.21–185.2, *P* = 0.034).

CD8+DR+ percentages were not significantly correlated with CRP (*R*: +0.40, *P* = 0.15), rheumatoid factor (*R*: 0.39, *P* = 0.17), or ESR (*R*: −0.030, *P* = 0.91).

## 4. Discussion

In this study, we aimed to explore whether there was an association between levels of complement factors and of T-cell activation and clinical events. We took advantage of a long term followup of our patients in the clinical immunology unit of our hospital. In the baseline evaluation, we detected lower C3 and C4 levels in patients with high titers of aCL or triple positivity. This association supports the hypothesis of complement activation in thrombotic APS. More interestingly, in the prospective follow-up phase of the study, lower C3 complement levels and increased CD8+DR+ T-cell values were risk factors for the development of APS-related clinical complications. Even if our study was performed in few patients, higher CD8+DR+ T-cell percentages were an independent risk factor for development of complications. Poor pregnancy outcomes have been described among primary APS pregnancies with hypocomplementemia [19]. The percentage of circulating CD8+DR+ T-cells has been suggested as a biological marker which accurately reflects disease activity in SLE patients [20]. Given the lack of useful biomarkers to assess the risk of clinical progression in thrombotic APS patients, these parameters are good candidates for further evaluation in a future multicenter prospective study with greater number of patients and greater power to predict APS-related complications.

In the evaluation of the relationship of the biomarkers with thrombotic APS, we found lower concentrations of complement factors in patients with thrombotic APS as compared with healthy controls. Infections, injuries, and other types of biological stress can activate the complement system [2]. We excluded patients with known causes of low complement activity at the time of sample collection. In our study, the complement activation status of these patients was similar to that observed in aPL positive patients with distinct autoimmune conditions who lacked APS clinical criteria and in SLE patients. In thrombotic APS, no association was detected between the presence of other tested autoantibodies and complement factors, suggesting that hypocomplementemia might be aPL dependent. It has been suggested that a proinflammatory state may coexist with thrombotic APS. In fact, accumulated evidence indicates that tissular ischemia or platelet aggregation may induce complement activation [8, 9]. The hypocomplementemia pattern that we observed affected C3 and C4, indicating that complement activation in thrombotic APS is produced mainly via the classic pathway. It was recently reported that patients with aPL had significantly increased levels of complement activation products [21]. Hypocomplementemia can be associated with increased immune complex formation. Oku et al. [7] found high immune complex levels in patients with primary APS. Carbone et al. [3] reported that 91% of women diagnosed with obstetric APS had high levels of circulating immune complex. Recently, complement activation affecting platelets was shown to be involved in vascular inflammation and thrombosis [22]. Interestingly, in our study, the relationship between complement values and inflammatory markers was better observed in APS patients than in aPL positive and SLE controls. The role of complement-mediated inflammation in reproductive failure in APS and the potential role of the immune modulation of the complement system (i.e., by the use of complement inhibitor therapies) in future studies have been discussed in recent publications of the 14th International Congress on Antiphospholipid Antibodies Task Force Reports [23, 24].

A limitation of our study is that complement split products were not evaluated. These products, rather than C3 and C4 levels, reflect more accurately complement activation. Complement activation products (C3a, C5a, and C5b-9) are able to activate endothelial cells, which result in loss of their antiinflammatory and antithrombotic potential. Direct determination of complement split products should be included in future studies.

We previously described increased levels of activated T-cell subsets in women with obstetric APS [13]. We also reported that higher levels of activated T-cells were associated with thrombosis in patients with HIV disease [15]. Although we performed the immunophenotypic study in only a small subgroup of patients, the results suggest that thrombotic APS is also associated with increased levels of T-cell activation. This hyperactivation status might be associated with reactivity against beta-2-glycoprotein-I, which has been previously demonstrated in APS [25]. On the other hand, higher percentages of T-cell activated subsets in thrombotic APS patients were similar to that described in patients with other systemic autoimmune diseases including SLE [26]. This immunophenotypic pattern of immune activation in thrombotic APS patients involved a higher expression of HLA class II (HLA-DR) molecules on CD4 and CD8 lymphocytes. A distinctive characteristic of thrombotic APS patients was the presence of a normal ratio of CD4+ cells coexpressing the alpha chain of the IL-2 receptor (CD25) over CD4+ cells coexpressing CD38 as compared with healthy controls, but higher than that observed in both disease control groups. As CD38 expression by CD4+ cells has been associated with low IL-2 production [27], these later results support the hypothesis that a proinflammatory Th1 immunophenotypic profile is predominant in thrombotic APS patients [28].

An interesting finding was the observation of the highest coexpression of CD38 and HLA-DR on CD4+ cells in SLE patients in agreement with recent studies describing that increased CD38 expression in T-cells was more prevalent in clinically active SLE [29]. A bias for IL-13 secretion has been described in CD4+CD38+ cells [27]. Th2 cytokines are involved in promoting humoral immunity including induction of IgE synthesis and promoting MHC class II expression. Increased autoreactive IgE autoantibodies have been recently described in SLE patients [30].

## Figures and Tables

**Table 1:** Clinical characteristics of patients with thrombotic APS and controls.

Parameter	Thrombotic APS (*n* = 38)	aPL positive controls (*n* = 14)	SLE controls (*n* = 16)	Healthy controls (*n* = 52)	*P *
Age, y, mean (range)	50 (29–78)	43 (37–59)	42 (30–69)	48 (30–75)	0.17

Sex					
Men (%)	28.9	21.4	25	36.5	0.87
Women (%)	71.1	78.6	75	63.5	

Risk factors of thrombosis	15 (39)	0	0	0	—

Thrombotic event					
Stroke	7 (18.42%)	0	0	0	—
DVT	4 (10.53%)	0	0	0	—
CRVT	4 (10.53%)	0	0	0	—
Pulmonary embolism	3 (7.89%)	0	0	0	—
Hepatic vein thrombosis	1 (2.63%)	0	0	0	—
Thrombophlebitis	1 (2.63%)	0	0	0	—
Different combinations of thrombotic events	12 (31.58%)	0	0	0	—
Thrombotic events and miscarriage	4 (10.53%)	0	0	0	—
Other	2 (5.26%)	0	0	0	—
Baseline disease					
Lupus like disease	0	4 (28.6%)	0	0	—
Sjogren's syndrome	0	1 (7.1%)	0	0	—
Systemic vasculitis	0	1 (7.1%)	0	0	—
Rheumatoid arthritis	0	1 (7.1%)	0	0	—
Thrombocytopenia	9 (23.7%)	3 (21.4%)	4 (25%)	0	—
Cutaneous lupus	0	1 (7.1%)	0	0	—
UCTD	0	3 (21.4%)	0	0	—
Clinical and biological markers					
CRP (mg/dL)	2.24 ± 6.84	0.32 ± 0.34	1.47 ± 2.82	—	0.55
ESR (mm/hour)	20.5 ± 24.8	9.9 ± 9.9	14.3 ± 8.9	—	0.28
RF (IU/mL)	26.4 ± 20.4	75.4 ± 183.9	23.4 ± 11.9	—	0.42
SLEDAI index	—	—	2.3 ± 1.8	—	—

CRP: C-reactive protein; CRVT: central retinal vein thrombosis; DVT: deep vein thrombosis; ESR: erythrocyte sedimentation rate; RF: rheumatoid factor; UDCD: undifferentiated connective-tissue disease.

**Table 2:** Concentration and functional activity of complement proteins in patients with thrombotic APS and healthy controls.

Parameter	Patients	aPL positive controls	SLE controls	Healthy controls	*P *
	(*n* = 38)	(*n* = 14)	(*n* = 16)	(*n* = 52)	
C3, mean ± SD mg/dL (range)	97.7 ± 26.3 (58–156)	108.8 ± 39 (61–202)	77 ± 20 (27–106)	113.4 ± 40.6 (61–305)	0.35^a^, 0.05^b^, 0.04^c^
Low C3 (%)^1^	41.2	30.8	43.8	19.2	0.74^a^, 0.76^b^, 0.04^c^
C4, mean ± SD mg/dL (range)	16.9 ± 8.5 (2–41)	21 ± 7 (12–35)	15 ± 6 (5–27)	23.9 ± 9.6 (12.8–74.4)	0.19^a^, 0.58^b^, 0.001^c^
Low C4 (%)^2^	63.9	38.5	56.3	17.3	0.14^a^, 0.77^b^, <0.01^c^
FB, mean ± SD mg/dL (range)	33 ± 7.7 (14–46.1)	31 ± 8 (21.9–47.1)	28 ± 6 (23–34)	31.0 ± 8.27 (20–63)	0.83^a^, 0.45^b^, 0.28^c^
Low FB (%)^3^	3.33	0	0	0	0.54^a^, 0.83^b^, 0.37^c^
CH100, mean ± SD Units/mL (range)	46.9 ± 17.9 (20–70)	60 ± 27 (23–89)	63 ± 24 (36–102)	70.5 ± 31.5 (20–165)	0.19^a^, 0.23^b^, 0.01^c^
Low CH100 (%)^4^	64.3	57.1	71.4	59.4	0.37^a^, 0.63^b^, 0.51^c^

APS: antiphospholipid syndrome; aPL positive controls: aPL+ patients without APS clinical criteria; HC: healthy controls; FB: factor B; ^1^C3 normal range: 83–172 mg/dL; ^2^C4 normal range: 17–51 mg/dL; ^3^FB normal range: 19–50 mg/dL; ^4^CH100 normal range: >70 units/mL. ^a^ANOVA comparison of thrombotic APS with aPL+ controls, ^b^ANOVA comparison of thrombotic APS with SLE controls, and ^c^ANOVA comparison of thrombotic APS with healthy controls.

**Table 3:** Serum concentration and functional activity of complement proteins in thrombotic APS patients with specific laboratory and clinical abnormalities.

Parameter		C3 (mg/dL)	C4 (mg/dL)	FB (mg/dL)	CH100 (U/mL)
aCL titer	High	87.6 ± 21.1	14.1 ± 7.7	30 ± 10.1	35.2 ± 23.7
	Medium	108.3 ± 28.6	20.7 ± 8.8	33.5 ± 7.3	48 ± 13.1
	*P *	0.027	0.026	0.32	0.31

ANA	Positive	84.3 ± 27.5	16.1 ± 11.9	27.3 ± 12.6	35.4 ± 24.8
	Negative	103.2 ± 24.2	17.13 ± 7.1	33.9 ± 7.7	37.8 ± 21.22
	*P *	0.54	0.76	0.09	0.85

Anti-DNA	Positive	86.6 ± 22.2	11.9 ± 6.1	34 ± 5	24.8 ± 3.8
	Negative	100 ± 26.9	18.1 ± 8.6	31.6 ± 10.3	41.8 ± 24.1
	*P *	0.26	0.08	0.62	0.19

Thrombocytopenia	Yes	90.9 ± 28.6	14.95 ± 8.3	25.6 ± 13.8	15.3 ± 13.6
	No	99.4 ± 25.9	17.4 ± 8.6	33.5 ± 7.9	42.8 ± 19.8
	*P *	0.46	0.48	0.07	0.046

Risk factors for thrombosis	Yes	97.5 ± 27.9	17.1 ± 8.9	33.4 ± 7.9	31.7 ± 24
	No	106 ± 28.2	19.3 ± 5.5	32.9 ± 8.9	47.0 ± 15.6
	*P *	0.526	0.55	0.89	0.42

High-titer aCL, IgG, or IgM anticardiolipin antibodies >80 U/mL. High-titer antinuclear antibodies (ANA) >1/160. High anti-DNA >20 IU/mL. Thrombocytopenia, platelet count < 100,000/*μ*L. Risk factors include arterial hypertension, portal hypertension, atrial fibrillation, dyslipidemia, homocysteinemia, hypergammaglobulinemia, and smoking.

**Table 4:** Bivariate correlation between complement levels and lymphocyte subsets in thrombotic APS patients.

	C3	C4	Factor B	CH100
CD4+ DR+	−0.23 (0.45)	−0.48 (0.09)	−0.55 (0.12)	0.06 (0.84)
CD4+ CD38+ DR+	0.02 (0.94)	−0.03 (0.94)	−0.51 (0.16)	0.23 (0.47)
CD4+ CD25+ DR+	−0.17 (0.58)	−0.27 (0.36)	−0.52 (0.16)	−0.04 (0.91)
CD8+ DR+	−0.12 (0.70)	−0.54 (0.05)	−0.42 (0.26)	−0.37 (0.23)

Pearson's correlation.

## References

[1] Miyakis S, Lockshin MD, Atsumi T (2006). International consensus statement on an update of the classification criteria for definite antiphospholipid syndrome (APS). *Journal of Thrombosis and Haemostasis*.

[2] Ricklin D, Lambris JD (2013). Complement in immune and inflammatory disorders: pathophysiological mechanisms. *Journal of Immunology*.

[3] Carbone J, Orera M, Rodríguez-Mahou M (1999). Immunological abnormalities in primary APS evolving into SLE: 6 years follow-up in women with repeated pregnancy loss. *Lupus*.

[4] Sugiura-Ogasawara M, Nozawa K, Nakanishi T, Hattori Y, Ozaki Y (2006). Complement as a predictor of further miscarriage in couples with recurrent miscarriages. *Human Reproduction*.

[5] Alijotas-Reig J (2010). The complement system as a main actor in the pathogenesis of obstetric antiphospholipid syndrome. *Medicina Clinica*.

[6] Romay-Penabad Z, Liu X X, Montiel-Manzano G, De Martínez EP, Pierangeli SS (2007). C5a receptor-deficient mice are protected from thrombophilia and endothelial cell activation induced by some antiphospholipid antibodies. *Annals of the New York Academy of Sciences*.

[7] Oku K, Atsumi T, Bohgaki M (2009). Complement activation in patients with primary antiphospholipid syndrome. *Annals of the Rheumatic Diseases*.

[8] Woollard KJ (2013). Immunological aspects of atherosclerosis. *Clinical Science*.

[9] Falk E, Nakano M, Bentzon JF, Finn AV, Virmani R (2013). Update on acute coronary syndromes: the pathologists’ view. *European Heart Journal*.

[10] Bruserud O, Lundin K (1987). The effect of drugs used in anticoagulation therapy on T lymphocyte activation in vitro. II. Warfarin inhibits T lymphocyte activation. *Journal of Clinical and Laboratory Immunology*.

[11] Girardi G (2005). Heparin treatment in pregnancy loss: potential therapeutic benefits beyond anticoagulation. *Journal of Reproductive Immunology*.

[12] Kolev M, Le Friec G, Kemper C (2013). The role of complement in CD4+ T cell homeostasis and effector functions. *Seminars in Immunology*.

[13] Carbone J, Gallego A, Lanio N (2009). Quantitative abnormalities of peripheral blood distinct T, B, and natural killer cell subsets and clinical findings in obstetric antiphospholipid syndrome. *Journal of Rheumatology*.

[14] Carbone J, Gallego A, Lanio N, Chean C, Navarro J, Sarmiento E (2010). Peripheral blood CD8+DR+ T-cell count: a potential new immunologic marker of unexplained recurrent abortion. *Fertility and Sterility*.

[15] Carbone J (2007). Immune activation and increased prevalence of thrombosis in HIV infection. *Journal of Acquired Immune Deficiency Syndromes*.

[16] Hochberg MC (1997). Updating the American College of Rheumatology revised criteria for the classification of systemic lupus erythematosus. *Arthritis and rheumatism*.

[17] Alarcón GS, Cianfrini L, Bradley LA (2002). Systemic lupus erythematosus in three ethnic groups. X: measuring cognitive impairment with the cognitive symptoms inventory. *Arthritis Care and Research*.

[18] Gladman DD, Goldsmith CH, Urowitz MB (1994). Sensitivity to change of 3 systemic lupus erythematosus disease activity indices: international validation. *Journal of Rheumatology*.

[19] Reggia R, Ziglioli T, Andreoli L (2012). Primary anti-phospholipid syndrome: any role for serum complement levels in predicting pregnancy complications?. *Rheumatology*.

[20] Viallard JF, Bloch-Michel C, Neau-Cransac M (2001). HLA-DR expression on lymphocyte subsets as a marker of disease activity in patients with systemic lupus erythematosus. *Clinical and Experimental Immunology*.

[21] Breen KA, Seed P, Parmar K, Moore GW, Stuart-Smith SE, Hunt BJ (2012). Complement activation in patients with isolated antiphospholipid antibodies or primary antiphospholipid syndrome. *Thrombosis and Haemostasis*.

[22] Lood C, Eriksson S, Gullstrand B (2012). Increased C1q, C4 and C3 deposition on platelets in patients with systemic lupus erythematosus-a possible link to venous thrombosis?. *Lupus*.

[23] de Jesus GR, Agmon-Levin N, Andrade CA (2014). 14th International Congress on Antiphospholipid Antibodies: task force report on obstetric antiphospholipid syndrome. *Autoimmunity Reviews*.

[24] Erkan D, Aguiar CL, Andrade D (2014). 14th International Congress on Antiphospholipid Antibodies: task force report on antiphospholipid syndrome treatment trends. *Autoimmunity Reviews*.

[25] Torres-Aguilar H, Blank M, Kivity S (2012). Tolerogenic dendritic cells inhibit antiphospholipid syndrome derived effector/memory CD4+ T cell response to *β*2GPI. *Annals of the Rheumatic Diseases*.

[26] Erkan D, Willis R, Basra G (2014). A prospective open-label pilot study of fluvastatin on proinflammatory and prothrombotic biomarkers in antiphospholipid antibody positive patients. *Annals of the Rheumatic Diseases*.

[27] Scalzo-Inguanti K, Plebanski M (2011). CD38 identifies a hypo-proliferative IL-13-secreting CD4+ T-cell subset that does not fit into existing naive and memory phenotype paradigms. *European Journal of Immunology*.

[28] Karakantza M, Theodorou GL, Meimaris N (2004). Type 1 and type 2 cytokine-producing CD4+ and CD8+ T cells in primary antiphospholipid syndrome. *Annals of Hematology*.

[29] Pavón EJ, Zumaquero E, Rosal-Vela A (2013). Increased CD38 expression in T cells and circulating anti-CD38 IgG autoantibodies differentially correlate with distinct cytokine profiles and disease activity in systemic lupus erythematosus patients. *Cytokine*.

[30] Dema B, Pellefigues C, Hasni S (2014). Autoreactive IgE is prevalent in systemic lupus erythematosus and is associated with increased disease activity and nephritis. *PLoS ONE*.

